# ^18^F-FDG PET intensity correlates with a hypoxic gene signature and other oncogenic abnormalities in operable non-small cell lung cancer

**DOI:** 10.1371/journal.pone.0199970

**Published:** 2018-07-02

**Authors:** Brendan T. Heiden, Guoan Chen, Matthew Hermann, Richard K. J. Brown, Mark B. Orringer, Jules Lin, Andrew C. Chang, Philip W. Carrott, William R. Lynch, Lili Zhao, David G. Beer, Rishindra M. Reddy

**Affiliations:** 1 Department of Surgery, Section of Thoracic Surgery, University of Michigan, Ann Arbor, MI, United States of America; 2 Department of Radiology, Division of Nuclear Medicine, University of Michigan, Ann Arbor, MI, United States of America; 3 Biostatistics Unit, University of Michigan Comprehensive Cancer Center, University of Michigan, Ann Arbor, MI, United States of America; University of Nebraska Medical Center, UNITED STATES

## Abstract

**Background:**

^18^F-fluorodeoxyglucose positron emission tomography (FDG-PET) is critical for staging non-small-cell lung cancer (NSCLC). While PET intensity carries prognostic significance, the genetic abnormalities associated with increased intensity remain unspecified.

**Methods:**

NSCLC samples (N = 34) from 1999 to 2011 for which PET data were available were identified from a prospectively collected tumor bank. PET intensity was classified as mild, moderate, or intense based on SUVmax measurement or radiology report. Associations between genome-wide expression (RNAseq) and PET intensity were determined. Associations with overall survival were then validated in two external NSCLC cohorts.

**Results:**

Overall survival was significantly worse in patients with PET-intense (N = 11) versus mild (N = 10) tumors (p = 0.039). Glycolytic gene expression patterns were markedly similar between intense and mild tumors. Gene ontology analysis demonstrated significant enhancement of cell-cycle and proliferative processes in FDG-intense tumors (p<0.001). Gene set enrichment analysis (GSEA) suggested associations between PET-intensity and canonical oncogenic signaling pathways including *MYC*, *NF-κB*, and *HIF-1*. Using an external cohort of 25 tumors with PET and genomic profiling data, common genes and gene sets were validated for additional study (P<0.05). Of these common gene sets, 20% were associated with hypoxia or HIF-1 signaling. While *HIF-1* expression did not correlate with poor survival in the NSCLC validation cohort (N = 442), established targets of hypoxia signaling (*PLAUR*, *ADM*, *CA9*) were significantly associated with poor overall survival.

**Conclusions:**

PET-intensity is associated with a variety of oncogenic alterations in operable NSCLC. Adjuvant targeting of these pathways may improve survival among patients with PET-intense tumors.

## Introduction

Lung cancer continues to be the number one cause of cancer-related deaths in the developed world [1]. Even when diagnosed at an early stage, patients still have high rates of recurrence. The most common histology of lung cancer is non-small-cell lung cancer (NSCLC) which accounts for approximately 85% of cases; of NSCLC, the most common pathologies are adenocarcinoma (40%), squamous cell carcinoma (30%), and large-cell carcinoma (10%) [2]. ^18^F-2-deoxy-D-glucose (FDG) positron emission tomography (PET scan) has become a standard tool for determining the operative candidacy of individuals with lung cancer by evaluating for the presence of regional or systemic metastases. FDG is a fluorescent glucose analog that accumulates at sites with elevated glucose metabolism, including many tumors. It has been well-established that the degree of FDG uptake by NSCLC tumors (i.e. the intensity) predicts survival, even among early stage surgical candidates [3–9].

PET imaging provides a unique insight into tumor cell metabolism. Many signaling pathways have been implicated in altering cancer cell metabolism, perhaps the most common being the phosphoinositide-3-kinase (PI3K) and HIF1 pathways [10–13]. HIF1 has been shown to activate pyruvate dehydrogenase kinases (PDKs), inhibiting the entry of pyruvate into the mitochondria for oxidative phosphorylation [14–16]. MYC–which interacts with HIF1 –has been shown to regulate many glycolytic enzymes, including GLUT transporters, lactate dehydrogenase (LDH), and PDK1 [17]. Indeed, a direct clinical correlation between *MYC* amplification and PET intensity has been demonstrated in human breast cancer [18]. A similar study in lung cancer by Nair *et al*. showed an association between PET intensity and *NF-κB* expression [19,20].

Despite this, the genetic abnormalities associated with increased PET intensity remain marginally understood. This study–which to our knowledge is the largest PET radiogenomic analysis to date–aims to better characterize the genomic alterations associated with increased FDG uptake in early stage lung cancers.

## Material and methods

### Patients and samples

Lung cancer tissues were collected from patients undergoing curative lung cancer surgery as part of a prospectively collected tissue bank beginning in 1991 through the present at the University of Michigan Health System. Thirty-four patients who had documented PET scans and resected non-small cell lung cancer between 1999 and 2011, for whom relevant tissue samples were available, were identified. Written consent was received and the project was approved by the local Institutional Review Board (Genetic Alterations in Human Lung Cancer IRBMED #1993–0215 (HUM00037727)). Patients were imaged by PET/CT (N = 17) or PET (N = 17) in accordance with standard protocol. All imaging occurred prior to operation; patients had not received neoadjuvant treatment. Primary tumor and adjacent non-neoplastic samples were obtained at the time of pulmonary resection, prepared and stored at -80C until use. Samples were examined histologically to identify regions with at least 60% cellularity for analysis. RNA was extracted and RNA-Seq transcriptome profiling was performed as described previously [21]. Genetic data is available in supplementary materials. Additional clinical information was collected from the electronic medical records and staging was performed according to the revised 7th TNM classification criteria [22]. Pathologic data including size, lymph node positivity, and histology were extracted from the final pathology report.

A PET validation cohort of lung cancers with similar patient demographics and pathology was identified and both radiologic and transcriptome data were obtained from Nair *et al*. [19]. All patient demographics were extracted from the raw data files that were obtained. A publicly available NSCLC cohort with similar patient demographics to the other two cohorts was used for prognostic validation of specific genes [21].

### FDG-PET imaging analysis

All scans were read prior to surgery and reports were available for review (N = 34). Where available, FDG-PET images were read again in the setting of this study and were quantified by maximum standard uptake values (SUV_max_) by two nuclear medicine radiologists (attending and senior resident with training in nuclear medicine); all studies were reviewed by the two radiologists together and a verbal agreement was achieved on tumor location and SUV intensity measurement. Images were grouped into mild, moderate, or intense FDG-uptake based on image analysis (N = 18, SUVmax lower cutoff 3.7; upper cutoff 8.6) and/or radiology reports (N = 16, qualitative). Genetic analysis was performed comparing FDG-intense (N = 11) and mild (N = 10) groups; moderate intensity tumors were excluded from the initial analysis (as done in prior studies to isolate extremes in gene expression) [19,20]. Tumor intensity was correlated with RNA expression profiles. For the PET validation cohort, tumors were grouped into tertiles based on SUVmax (SUVmax lower cutoff 2.7, N = 8; upper cutoff 6.3, N = 8). The SUV_max_ cutoffs for the study and validation cohorts were not pre-specified but instead data-driven tertiles, consistent with prior reports [4,19,20].

### Gene expression

Single gene enrichment was determined using unpaired T-test with a fold-change cutoff for both study and PET validation cohort (fold change>2.0, p<0.05); significance analysis of microarrays (SAM) was also performed to further control for multiple comparisons [23]. To determine potential underlying biological processes associated with high PET-intensity correlated genes, gene ontology enrichment analysis was performed based on the 100 most significantly enriched genes using the DAVID bioinformatics platform [24]. To examine specific signaling pathways, including metabolic and oncogenesis pathways, samples were further analyzed by Gene Set Enrichment Analysis (GSEA) [25]. Over 4,700 gene sets were assessed for enrichment using the MSigDB database; further, the Kyoto Encyclopedia of Genes and Genomics (KEGG) was used to interrogate 195 metabolic pathways within our data pool. Pathways that were significantly differentiated between FDG-intense and mild groups were reported (FDR<0.25; p<0.05).

### Statistical analysis

Data were analyzed using GraphPad Prism 6 (GraphPad software), Microsoft Excel, and R software (v 3.2.1). Individual genes and gene sets were validated using an external cohort of patients. Overlapping genetic abnormalities between the two data pools were identified and reported based on (1) individual genes (fold change>2, p<0.05) and (2) GSEA (FDR<0.25; p<0.05). All survival curves were constructed using the Kaplan-Meier method in R software, and survival differences were assessed by the log-rank test using the median gene expression as cutoff value. The primary outcome was overall survival, censored at five years. Patients were followed from May 1999 through January 2016.

Multivariate analysis for gene expression and overall survival (controlling for gender, age, race, stage, and smoking history) was performed using the PHREG procedure (SAS).

## Results

### Patient demographics

Tumors from thirty four patients with early stage non-small cell lung cancer (NSCLC) were selected for the study cohort. The mean patient age was 70 years (Table 1). Tumors were predominantly stage I-II (30/34, 88%) and a vast majority had adenocarcinoma histology (26/34, 76%). The median time to operation from PET acquisition was 44 days. The PET validation cohort included 25 early stage NSCLC tumors. The mean patient age in this cohort was 71 years. Most tumors were stage I-II (23/25, 92%) and displayed adenocarcinoma histology (20/25, 80%). The median time between PET acquisition and operation was 27 days(19). The prognostic validation cohort was used to examine the prognostic significance of individual genes identified through the radiogenomic analysis. This cohort included 442 lung adenocarcinoma tumors, 84% (374/442) of which were stage I-II. The mean patient age was 64 years.

**Table 1:** Patient demographics.

Characteristics	Study (N = 34)	PET Validation (N = 25)^a^	Prognostic Validation^b^ (N = 442)
**Mean Age** **(intq. range), y**	**70 (62–77)**	**71 (64–77)**	**64 (N/A)**
**Sex (%)**			
** Male**	**19 (55)**	**18 (72)**	**223 (50)**
** Female**	**15 (45)**	**7 (28)**	**219 (50)**
**Stage (%)**			
** I-II**	**30 (88)**	**23 (92)**	**374 (84)**
** III-IV**	**4 (12)**	**2 (8)**	**68 (16)**
**Histology (%)**			
** Adenocarcinoma**	**26 (76)**	**20 (80)**	**442 (100)**
** Other**	**8 (24)**	**5 (20)**	**0 (0)**

^a^Nair *et al*. 2012

^b^Shedden *et al*. 2008

N/A, not available; PET, positron emission tomography; y, years

### Radiologic analysis

**Fig 1:**
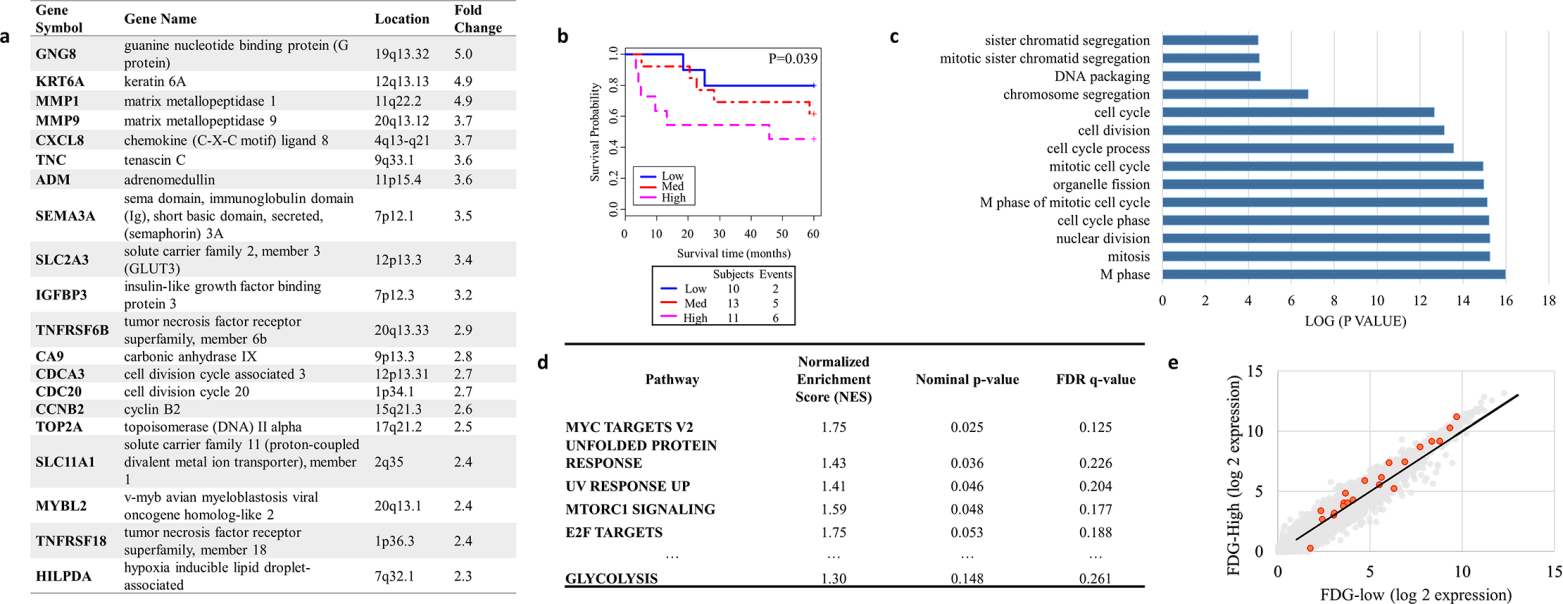
Genetic analysis of study group. (a) Selected genes upregulated in high PET intensity tumors (fold-change high vs. low >2, p<0.05). (b) Kaplan-Meier survivor curve representing overall survival (in months) for patients with high PET-intensity tumors (N = 11), medium intensity tumors (N = 13), and low intensity tumors (N = 10). (c) The most significantly enriched genes in PET-high tumors (p<0.05, fold change>2.0) were interrogated by DAVID gene ontology pathway analysis [24]. Significant functional groups are shown. P-values are quantified as log units. (d) Average rank-based GSEA results for MSigDB Hallmark pathways. (e) RNA levels of core glycolysis enzymes (red) versus all genes (gray) in PET high (y axis) versus low (x-axis) tumors. “Core” enzymes were labeled such according to the KEGG gene set database.

Tumors were classified as “high” or “low” based on observed SUV intensity or radiology reports. Patients with high SUV intensity tumors had a significantly worse overall 5 year survival than those with low intensity tumors (p = 0.039, Fig 1B), consistent with prior literature [3]. Representative scans are shown in supplementary figure (S3 Fig). Additional patient characteristics, including smoking history, and tumor features, including stage, histology, size, and location, are displayed in Table 2.

**Table 2:** Patient clinical characteristics.

PET Intensity	Age	Sex	Smoking (pack years)	Stage	T	N	M	Histology	Differentiation	Size (cm)	LN	Location
Low	31	F	none	Ib	2	0	0	Adenoid cystic carcinoma	N/A	2.5 x 2.0 x 1.5	0/11	RML
	72	M	50	IIIa	2	2	0	Adeno	Well	3.2 x 3.5 x 3.5	1/14	LUL
	46	F	47	Ia	1	0	x	Large cell	N/A	3 x 2.5 x 2.5	0/22	RUL
	73	M	30	Ia	1	0	0	Adeno	Well	2 x 2 x 2	0/9	LUL
	83	M	20	Ia	1	0	0	Adeno	Poor	1.5 x 1.2 x 0.9	0/16	LUL
	60	M	68	Ib	2	0	0	Adeno	Moderately poor	1.5	0/0	LUL
	73	M	55	Ib	2	0	0	Adeno	Well	3.0	0/4	LLL
	65	F	100	Ia	1	0	0	Adeno	Well	1.3	0/3	RUL
	62	F	50	Ia	1	0	0	Adeno	Moderate	3 x 2.5 x 2.0	0/13	LUL
	77	F	none	IIa	1	1	0	Adeno	Moderately well	1.6 x 1.6 x 1.2	1/3	LLL
High	63	F	68	Ia	1	0	0	Adeno	Poor	2.5 x 1.5 x 2.2	0/14	LUL
	76	M	60	IIIa	2	2	0	Adeno	Poor	6.7 x 4.5 x 3.5	4/14	RLL
	90	F	10	IIIb	4	2	0	Large cell	Well	4.5 x 4.3 x 2.5	10/20	RUL, RML
	72	M	40	Ib	2	0	0	Adeno	Moderately well	6.9 x 5.2 x 2.8	0/15	RUL
	72	F	60	IIb	2	1	0	Large cell	N/A	5 x 4 x 3	1/10	RLL
	77	M	18	Ib	2	0	0	Adeno	Moderately well	5 x 3 x 5	0/5	LUL
	62	F	42	IIb	2	1	0	Adeno	Moderate	2 x 2 x 3	1/7	LLL
	76	M	40	Ib	2	0	0	Adeno	Poor	5.2 x 4.4 x 4.0	0/6	LUL
	79	F	120	Ia	1	0	0	Adeno	Moderate	2.5 x 1.7 x 1.5	0/16	RUL
	77	F	40	Ib	2	0	0	Large cell	N/A	7.5 x 7.0 x 5.0	0/8	RLL
	71	F	54	IIb	3	0	0	Adeno	Moderate	9 x 7 x 5	0/34	RUL

Adeno, adenocarcinoma; LLL, left lower lobe; LN, lymph nodes; LUL, left upper lobe; N/A, not available; RLL, right lower lobe; RML, right middle lobe; RUL, right upper lobe

### Genomic and pathway analysis of study group

Individual genes that were significantly upregulated in the PET-intense tumors were further interrogated (p<0.05, fold change>2.0, Fig 1A). Upregulated genes included the *GLUT3* transmembrane glucose transporter, cell cycle proteins, and various matrix metalloproteases. These genetic changes did not appear to cluster to specific chromosomal loci. Analysis controlling for multiple comparisons is available in S1 and S2 Figs. Sequencing analysis of common NSCLC mutations was performed [25]. Interestingly, no difference was observed in the rate of *TP53*, *RAS*, or *EGFR* mutations between the high and low intensity tumor samples (S3 Fig). DAVID gene ontology analysis was next performed to determine potential underlying biological processes associated with high PET-intensity correlated genes. This revealed pathways that were highly associated with cell proliferation processes (Fig 1C). To further determine specific pathways associated with PET intensity, including glycolysis and other metabolic pathways, gene set enrichment analysis was performed (Fig 1D). This revealed MYC targets as potential correlates to PET-intensity. Since the glycolytic pathway was not significant by this analysis, we then examined individual glycolytic genes to determine if only one or two of these genes were overexpressed. Modest enrichment of multiple glycolytic genes was observed in high PET-intensity tumors (Fig 1E).

### Radiogenomic and prognostic validation

**Fig 2:**
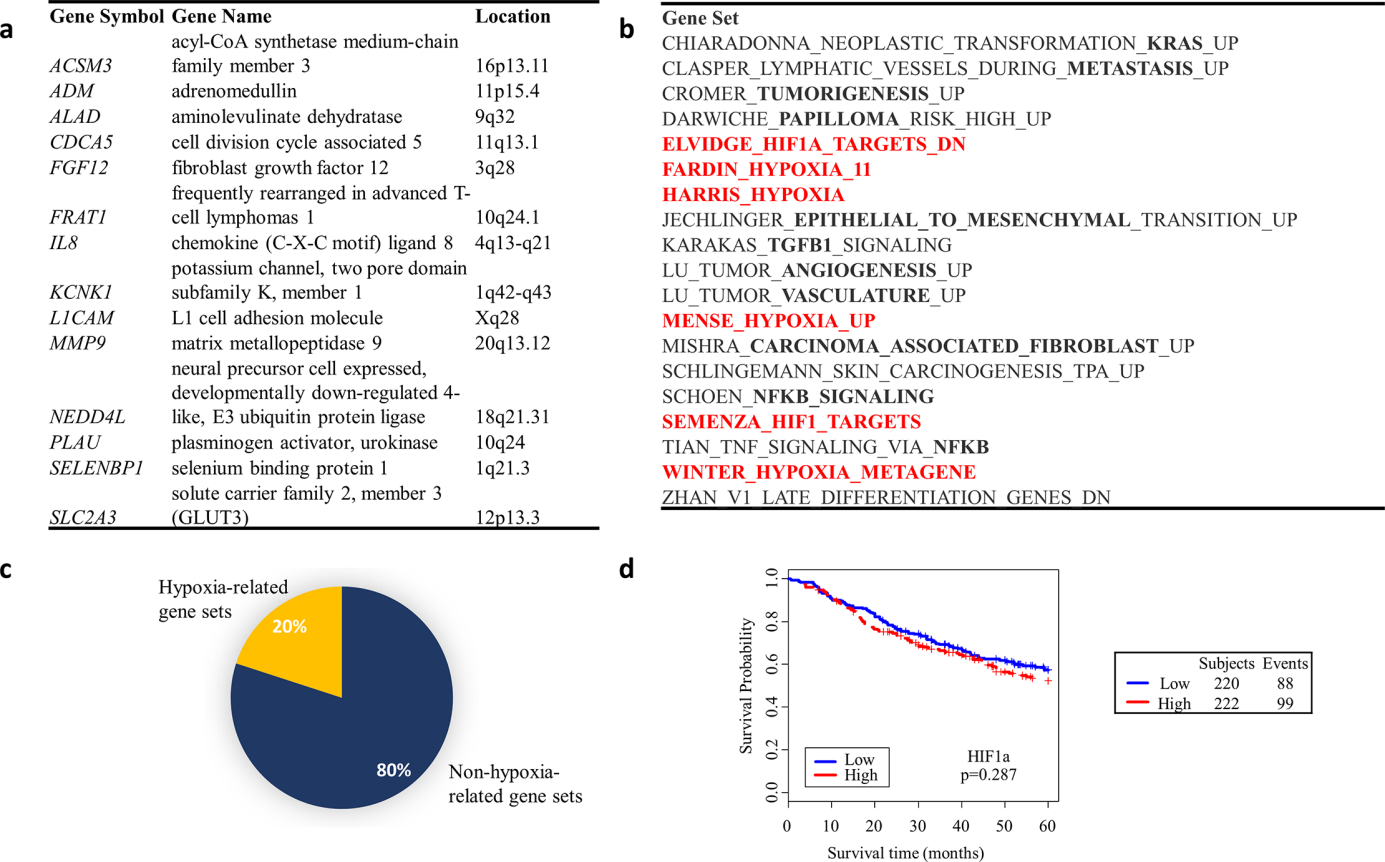
Validated radiogenomic abnormalities in two cohorts of patients with early-stage NSCLC. (a) The most significantly enriched genes in PET-intense tumors (p<0.05, fold change>2.0) were selected from the study and PET validation cohort. Selected genes that overlapped are displayed in the table. (b) Average rank-based GSEA results for all pathways in the MSigDB database that were enriched in high-intensity tumors in both the study and PET validation cohorts and (c) quantified relative to hypoxia. (d) Kaplan-Meier survivor curves and log-rank test of *HIF1A* expression in prognostic validation cohort (N = 442) using median gene expression as cutoff to divide low and high expression.

As the number of samples included in the initial study cohort was relatively small, a PET validation cohort was obtained to further confirm any significant genetic findings. Data from 25 patients with early stage NSCLC tumors for which both radiologic PET and genetic data were available were obtained from Nair *et al*.[19]. Genes that were significantly upregulated in the high intensity tumors from both the study and PET validation cohort included the *GLUT3* transmembrane glucose transporter, among others (p<0.05, fold change>2.0, Fig 2A). Gene set enrichment analysis revealed 30 common gene sets from the MSigDB database, including many canonical oncogenic signaling pathways (Fig 2B). Of these common gene sets, 20% involved hypoxia signaling (Fig 2C). We therefore hypothesized that *HIF1A* expression may correlate with increased PET-intensity and the observed inferior survival among these patients. However, *HIF1A* expression did not correlate with poor survival in the prognostic validation cohort (Fig 2D).

**Fig 3:**
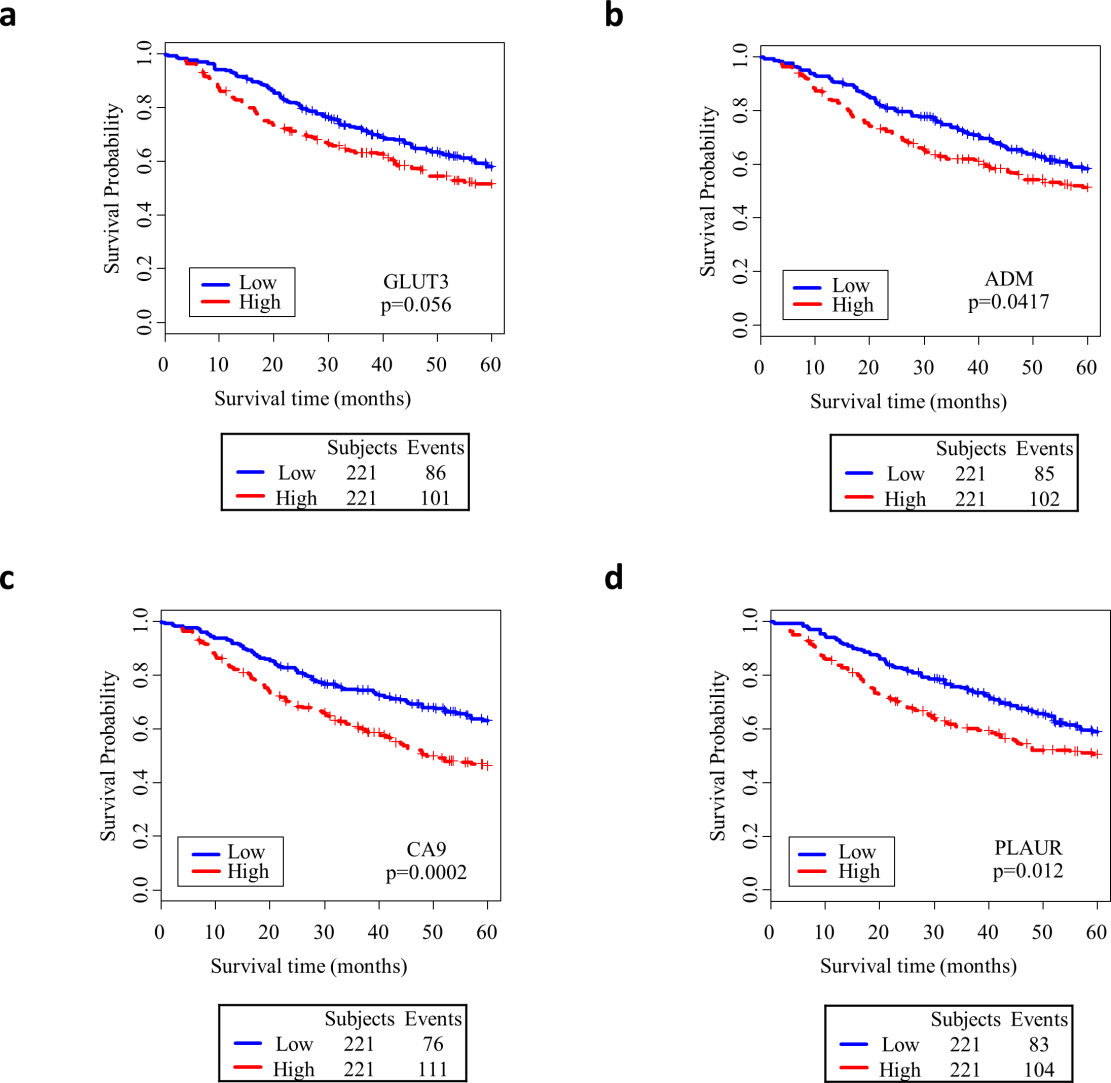
Kaplan-Meier survivor curves and log-rank test of selected targets of HIF signaling in prognostic validation cohort (N = 442) using median gene expression as cutoff to divide low and high expression. Downstream targets of HIF including (a) *GLUT3*, (b) *ADM*, (c) *CA9*, and (d) *PLAUR* were associated with worse survival. Genes were selected according to validated MSigDB gene sets related to hypoxia signaling.

Since *HIF1A* expression did not correlate with poor survival, canonical downstream targets of HIF1A signaling were selected from the MSigDB hallmark gene set for hypoxia signaling. While *HIF1A* transcript levels do not predict poor survival, elevated expression of its downstream targets, including *GLUT3*, *ADM*, *PLAUR*, and *CA9*, correlate with worse overall survival among NSCLC patients (Fig 3). Multivariate analysis controlling for sex, age, stage, smoking history, and race is included in S4 Fig.

## Discussion

This study was designed to identify the genetic abnormalities associated with increased PET intensity in operable NSCLC. Since it is well-established that high PET intensity portends a poor prognosis, understanding the genetic drivers of this phenomenon could be important from a therapeutic perspective. Our data suggest that a heterogeneous group of genetic irregularities may contribute to increased PET intensity. While this may suggest that avid glucose uptake is a convergence of many oncogenic abnormalities, hypoxia signaling seemed to stand out within our data analysis. This finding is interesting for a variety of reasons.

First, decades of research have proposed that tumors–even in the presence of oxygen–paradoxically rely on glycolysis as opposed to the more efficient mitochondrial oxidative phosphorylation for their metabolic needs (“Warburg Effect”) [26–28]. While the advantages of this metabolic adaptation are debated, many believe that the byproducts of glycolysis drive other important biosynthetic pathways, therefore providing a selective advantage to rapidly-dividing cancer cells [26,27]. Our data show that FDG-intense NSCLC tumors share gene signatures suggestive of hypoxia. It remains unclear if the FDG-intense tumor microenvironment is actually hypoxic, or instead, as the Warburg Effect suggests, if hypoxia signaling confers some sort of survival advantage within normoxic environments. If so, elevated FDG uptake may indeed be *in vivo* proof of the Warburg Effect in NSCLC.

Second, HIF-regulated genes include 9 of the 10 enzymes that function in glycolysis [13]. Surprisingly, and contrary to our original hypothesis, PET intensity does not appear to be influenced by differential expression of glycolysis genes. HIF signaling may instead mediate glycolysis-independent phenomena in these tumors. The seemingly underwhelming contribution of glycolytic gene variability is interesting nevertheless and deserves further study.

Finally, the downstream genetic changes induced by hypoxia in NSCLC seem to be associated with a worse prognosis among surgical patients. Our study identified CA9 and GLUT3 as genes with a prognostic significance. GLUT3 is a canonical glucose transporter native to many tissues including the brain; it has been implicated in NSCLC and has been associated with FDG intensity in more limited studies [29–31]. Targeting CA9, a carbonic anhydrase which detoxifies the gaseous byproducts of hypoxic metabolism, has demonstrated a potential therapeutic benefit in some cancers [15,32,33]. Indeed, a monoclonal antibody against CA9 (gerentuximab) has shown considerable promise in renal cell carcinoma and may be suitable for NSCLC patients with PET intense tumors [34]. Bioreductive prodrugs are also being studied as compelling therapeutic options. These drugs have the advantage of selectively targeting hypoxic areas and hence may have high tumor specificity [35]. Nevertheless, the clinical utility of HIF targeting remains poorly explored and warrants further study [33,36,37].

Perhaps the most pertinent implication of studies like these is that PET intensity predicts a group of NSCLC patients–who despite “curative surgery”–have a relatively poor overall survival (Fig 1) [3]. Our study identifies genome-wide targets that may be harnessed in an adjuvant or neoadjuvant setting to improve the outcome of this disadvantaged subset of patients. Certainly, as PET imaging becomes more affordable and standardized for monitoring tumor progression, this topic may be of further interest.

This study is limited by the number of patients. To compensate, two distinct cohorts were identified and analyzed separately. This study was also limited in its strength of radiographic data. It was not possible to quantify every PET image due to technical limitations. However, many physicians do not receive explicit SUV values in the radiology reports. Therefore, these findings are still generally applicable to physicians who are familiar with evaluating such imaging without quantification. Finally, the genetic analysis was limited due to the heterogeneity within the samples. Again, this may suggest that PET-intensity represents a convergence of many distinct genetic phenomena.

In conclusion, preoperative FDG-PET intensity in NSCLC patients correlates with worse survival. These data show that a genetic signature consistent with hypoxia correlates with high intensity tumors. Further research is warranted to determine the therapeutic and clinical significance of these findings.

## Supporting information

S1 Fig (a) Representative cross-sectional FDG-PET scan images of high and low intensity tumors. (b) Percent mutation of common NSCLC mutations including *TP53*, *EGFR*, and *KRAS* between high and low intensity tumors.
(TIF)

S2 Fig Significance Analysis of Microarray (SAM) results for PET study cohort. (TIF)

S3 Fig Significance Analysis of Microarray (SAM) results for PET validation cohort. (TIF)

S4 Fig Multivariate analysis for gene expression and overall survival (controlling for gender, age, race, stage, and smoking history). (TIF)

S1 File Raw file of RNA-seq data. (TIF)
