# Supplementary figures and images for: 18F-FDG PET intensity correlates with a hypoxic gene signature and other oncogenic abnormalities in operable non-small cell lung cancer
Source: PLoS One. 2018 Jul 2;13(7):e0199970. doi: 10.1371/journal.pone.0199970 (PMC6028077; doi:10.1371/journal.pone.0199970)

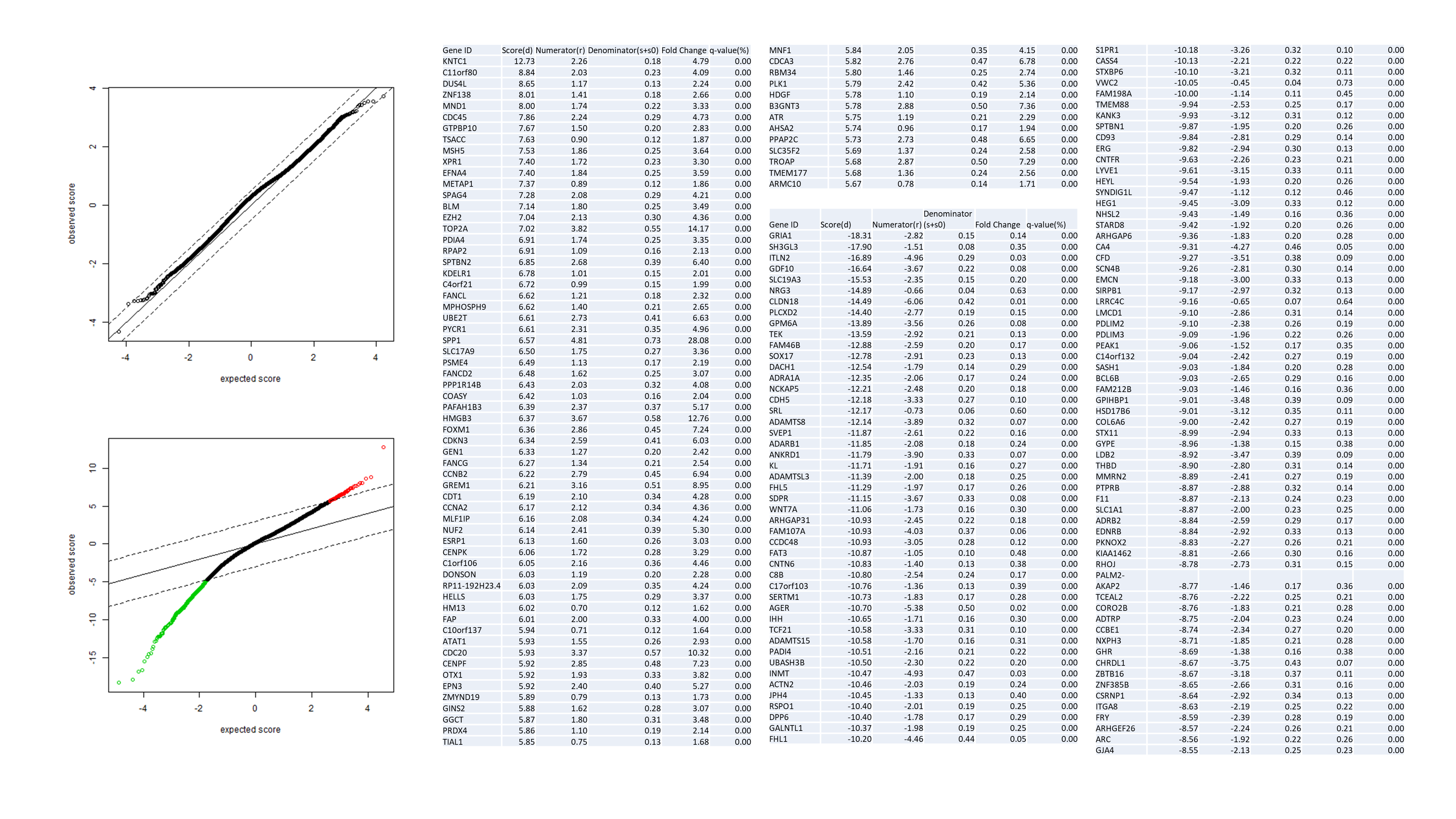

Supplement: S1 Fig — (a) Representative cross-sectional FDG-PET scan images of high and low intensity tumors. (b) Percent mutation of common NSCLC mutations including TP53, EGFR, and KRAS between high and low intensity tumors. (TIF) [file pone.0199970.s001.TIF]

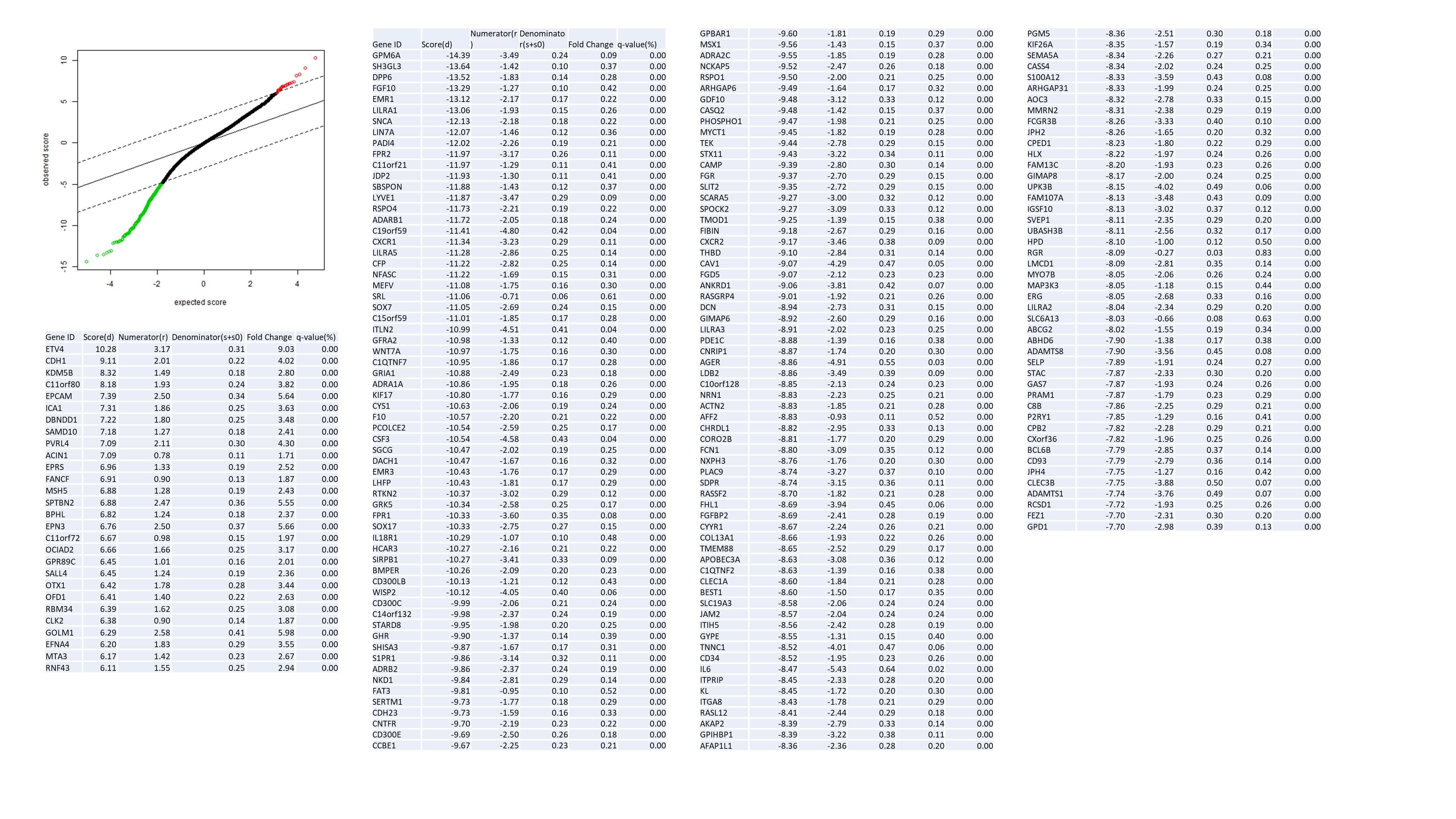

Supplement: S2 Fig — (TIF) [file pone.0199970.s002.TIF]

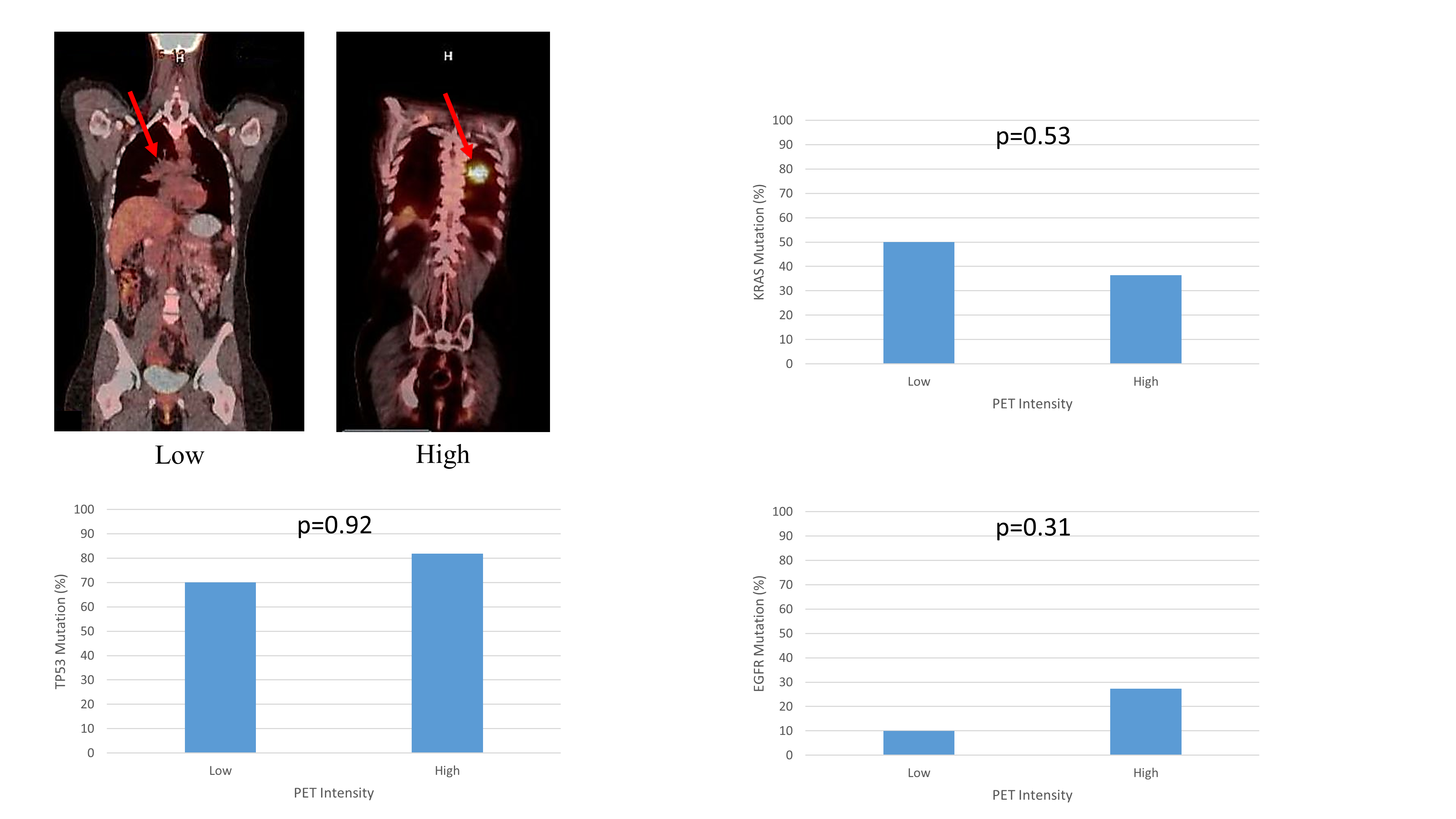

Supplement: S3 Fig — (TIF) [file pone.0199970.s003.TIF]

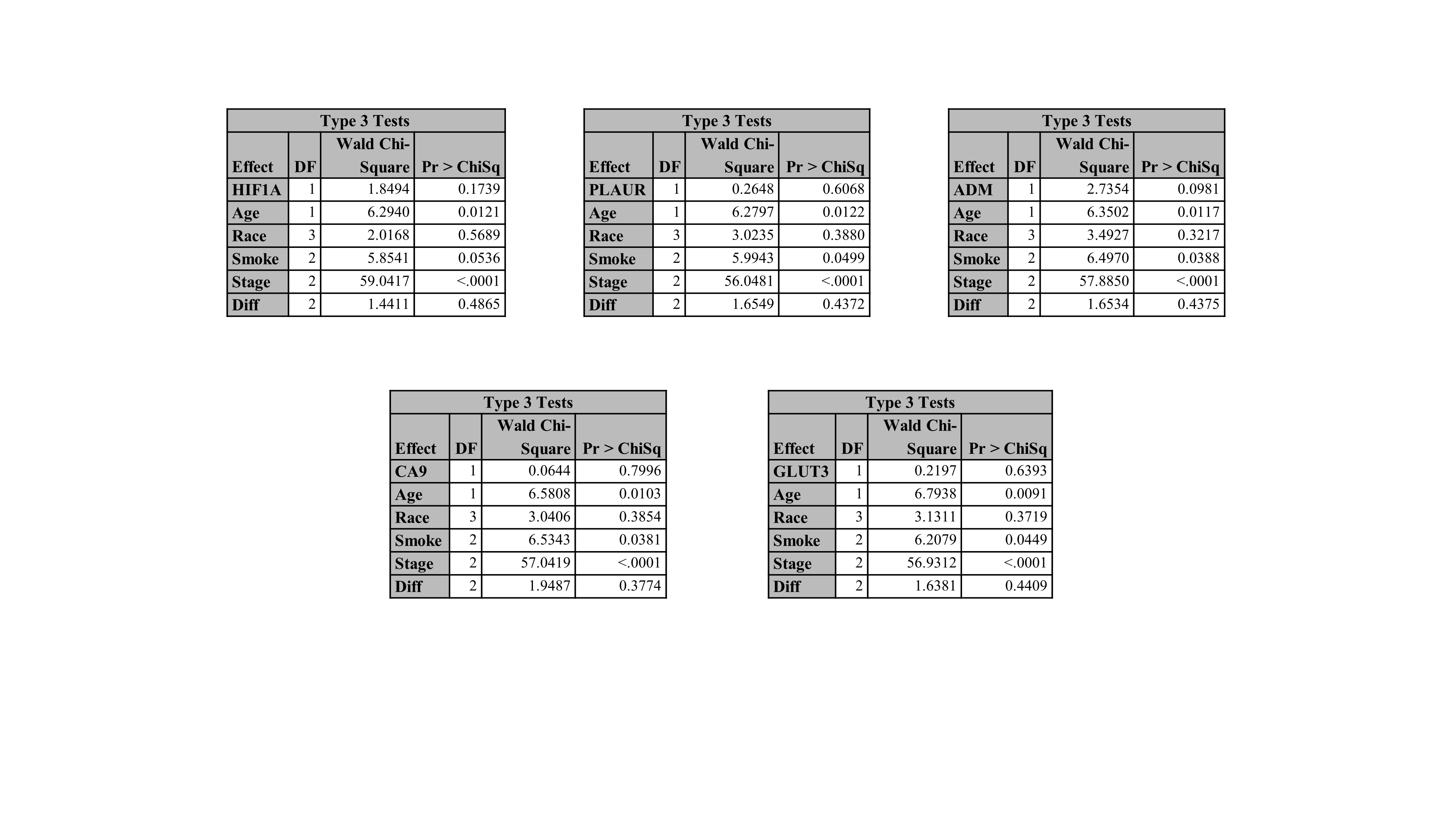

Supplement: S4 Fig — (TIF) [file pone.0199970.s004.TIF]
